# A scoping review comparing two common surgical approaches to the hip for hemiarthroplasty

**DOI:** 10.1186/s12893-019-0493-9

**Published:** 2019-03-08

**Authors:** James Fullam, Paraskevas G. Theodosi, John Charity, Victoria A. Goodwin

**Affiliations:** 1 NIHR CLAHRC South West Peninsula (PenCLAHRC), University of Exeter Medical School, Room 2.26, South Cloisters, St Luke’s Campus, Exeter, EX1 2LU UK; 2 Department of Trauma and Orthopaedics, Princess Elizabeth Orthopaedic Centre, Royal Devon and Exeter Hospital, Exeter, UK

**Keywords:** Hip fracture, Femoral neck fracture, Surgical approach, Posterior approach, Direct lateral approach

## Abstract

**Background:**

Hemiarthroplasty for hip fracture is a common surgical procedure. A number of distinct approaches are used to access the hip joint. The most commonly used are the direct lateral approach (DLA), and the posterior approach (PA). Internationally there is little consensus on which of these approaches to use. Current guidance is based on a limited selection of evidence and choice of approach is frequently based on surgeon preference. Historically, recommendations have been made based on dislocation rates. In light of technical advancements and greater recognition of patient priorities, outcomes such as post-operative function and pain may be considered more important in the modern context. The aim of this scoping review was to summarise the literature pertaining to the comparison of common surgical approaches to the hip for hemiarthroplasty.

**Methods:**

A scoping review methodology was used to examine the range and nature of primary research. Using systematic methods we searched for studies that directly compared the DLA and PA. Studies reporting the following outcomes were considered; dislocation, mortality, pain, activities of daily living, functionality, health-related quality of life, length of stay, surgeon assessment of difficulty, and adverse events. MEDLINE, EMBASE and The Cochrane Library were searched. Relevant information was extracted and synthesis of the retrieved data followed a basic content analytical approach.

**Results:**

A total of 13 studies were retrieved: 12 observational studies and 1 randomised trial. The majority of studies were based at single sites. Larger observational studies using multi-site and national registry data have emerged in recent years. Reporting of technique and outcomes is inconsistent. A trend for higher rates of dislocation using the PA was observed and eight studies recommended the use of the DLA over the PA.

**Conclusions:**

This scoping review demonstrates that the existing evidence is highly heterogeneous in nature and not of a sufficient quality to inform practice recommendations. This issue would be best addressed by additional RCTs, and high quality national-level observational data. Standardisation of the recording of patient risk factors, surgical and post-operative intervention protocols, and outcomes in all study designs would strengthen the potential for valid comparison of future findings.

## Background

The personal and wider social effects of hip fractures are profound. Over 4000 NHS beds are continuously occupied by hip fracture patients. Total UK annual hospital costs associated with hip fractures have been estimated at £1.1 billion [1]. White and Griffiths predict a trebling of inpatient costs from 2011 levels by 2033 in England alone, resulting both from higher prevalence of hip fracture, and the requirement for additional bed days based on increased perioperative morbidity [2]. Hemiarthroplasty is a common surgical procedure for treating displaced fractures of the femoral neck, the most prevalent type of hip fracture.

There are a number of distinct surgical approaches that may be used to access the hip joint. According to the few national registers that collect data on surgical approaches for hemiarthroplasty [3, 4], the direct lateral approach (DLA) and posterior approach (PA) are commonly used internationally. Anterior and anterolateral approaches are also used, but less often. Internationally it appears that choice of approach is frequently based on surgeon preference, as a result of training and experience, rather than rigid adherence to guidelines or evidence guided [5].

In the UK, practice is guided by both personal preference of surgeons, and a NICE guideline which recommends the anterolateral approach over a posterior approach [6]. However, the evidence informing the clinical guideline is limited, dated, and of poor quality. The evidence is drawn from only two studies, one RCT [7] and one cohort study [8], both of which have serious limitations in the context of informing a procedural guideline. In the face of emerging findings, it is unclear if guidance or predominating practice is still based on the best available evidence.

The classic PA is that developed by Austin Moore in the 1950s in the US, referred to as the Moore or Southern approach [9]. The main features of this approach are division of the short external rotators, piriformis and part of the quadratus femoris, while sparing the hip abductor musculature [10]. The approach provides good visualisation of the acetabulum and femur, and extensile exposure to both as required [11]. Theoretical advantages include lower risk of femoral shaft fracture, shorter operation time and reduced blood loss [12]. The posterior approach can be performed with or without repair of the posterior joint capsule and other muscle and tendon sparing modifications [13–15]. The PA is not without controversy. Abandonment of the PA in the context of hemiarthroplasties has been recommended due to the risk of postoperative dislocation [16]. This increased risk of dislocation that has been demonstrated in more contemporary observational studies [8, 17]. However, despite this, it is clear that surgeons continue to find the posterior approach to be of value. A recent study showing better patient reported outcomes, including quality of life, with the PA compared to the DLA in a large sample appear to provide some support for its continued use [18]. The DLA is characterised by division of the anterior portion of the gluteus medius and minimus muscles [19].

Hip fracture patients endure debilitating loss of function, recovery is complex and challenging [20]. Thirty-day mortality currently stands at around 7% but is expected to rise to 8.9–9.3% [2]. Observations confirm that the hip fracture population is increasingly frail, displays greater comorbidity, and more of these patients are living at home with higher dependency on social services [21]. Surgical approach in hemiarthroplasty is a contributing factor in operative success and regain of function.

## Methods

Considering the lack of consensus, and growing research output related to surgical approach for hip hemiarthroplasty, the authors propose that this is a pertinent time to explore the existing literature using a scoping review methodology. The aim of this scoping review was to summarise the literature pertaining to the comparison of common surgical approaches to the hip for hemiarthroplasty. The review sought to identify (1) critical knowledge gaps that contribute to a lack of consensus on surgical approach to hemiarthroplasty, and (2) how future research should be directed to address these gaps?

A scoping review is a framework-guided approach to reviewing research evidence. It may be used to: rapidly map the range and nature of literature in an area; to determine the value of undertaking a full systematic review; to identify gaps in the existing literature; and to set priorities for future research [22–24]. In contrast to a systematic review they allow for more broad research questions and permit the inclusion of different study designs. Quality appraisal of individual studies does not tend to fall within the remit of a scoping review, although some comment as to the overall quality of evidence may be offered [25].

The application of labels to the various approaches is inconsistent in the literature. For the purposes of this review, labels were applied in the following way. Any approach referencing a “Moore” or “Southern” approach was considered a PA. Approaches referencing a “Hardinge” approach were labelled as DLA. If a “Watson-Jones” type approach was referenced it was considered an anterolateral approach and not included in this review. This follows the classification system of Onyemaechi et al. [5], and various surgical textbooks.

### Search strategy

A refined search strategy was developed with the assistance of an information specialist. A search strategy optimised for identifying studies using a posterior approach was employed as this is the approach with the most consistent and accurate labelling. MEDLINE and EMBASE in Ovid were searched using the appropriate MeSH and EMTREE headings and subheadings, supplemented with free text. The Cochrane Library was also searched using an adapted search strategy. Searches were carried out in March 2017. Before optimisation for specific databases, search terms and strings would have approximated the following; hip* or hip fractur* or femoral neck fractur* or displaced intracapsular AND hemiarthoplast* AND posterior. To be eligible for inclusion retrieved studies were subsequently scanned to confirm a direct comparison with a DLA. Studies published prior to 1980 were not eligible for inclusion in this review, as it is probable that patients were subject to rehabilitation protocols that would be considered obsolete today. Only studies published in English were included. The reference lists of retrieved studies, relevant guidelines and previous reviews were scanned for relevant material. Retrieved citations were collated in Endnote × 7.1. Duplicate citations were removed.

### Selection process

Preliminary title screening was carried out by one researcher. Following this, two researchers independently reviewed abstracts for inclusion. Reviewers discussed challenges throughout the selection process. Following abstract review, full text articles were independently reviewed by two researchers for final inclusion. Where disagreements on study inclusion occurred, a third independent reviewer was consulted. Studies were included if the primary aim of the study was the direct comparison of common surgical approaches for hemiarthroplasty of the hip. At least one patient or surgical outcome must have been reported. Observational and experimental study designs were eligible for inclusion. Studies were excluded if: they could not be retrieved in an English full-text version; they were published prior to 1980; the study was a case report(s) (5 patients or less), a patient group was operated on using an alternative surgical approach, e.g. medial or direct anterior approach. Studies in which surgical approach was reported and considered as a potential factor affecting outcomes, but not the focus of the study (e.g. studies comparing cemented and uncemented hemiarthroplasties, or studies comparing unipolar versus bipolar hemiarthroplasties) were excluded.

Relevant information was extracted using a data extraction chart developed by the authors, and guided by recommendations of scoping review methodologists [23, 26]. The following data was extracted: year and setting of the study; details of the surgical approaches utilised; outcomes; data referring to dislocation rates, and the approach recommended according to the findings of individual studies if reported. Synthesis of the retrieved data followed a basic content analytical approach. The focus was to critically conceptualise the overarching features of the available evidence; with a view to highlighting and assessing its relevance to current and future practice, and identifying needs that can be addressed by future research.

## Results

**Fig. 1:**
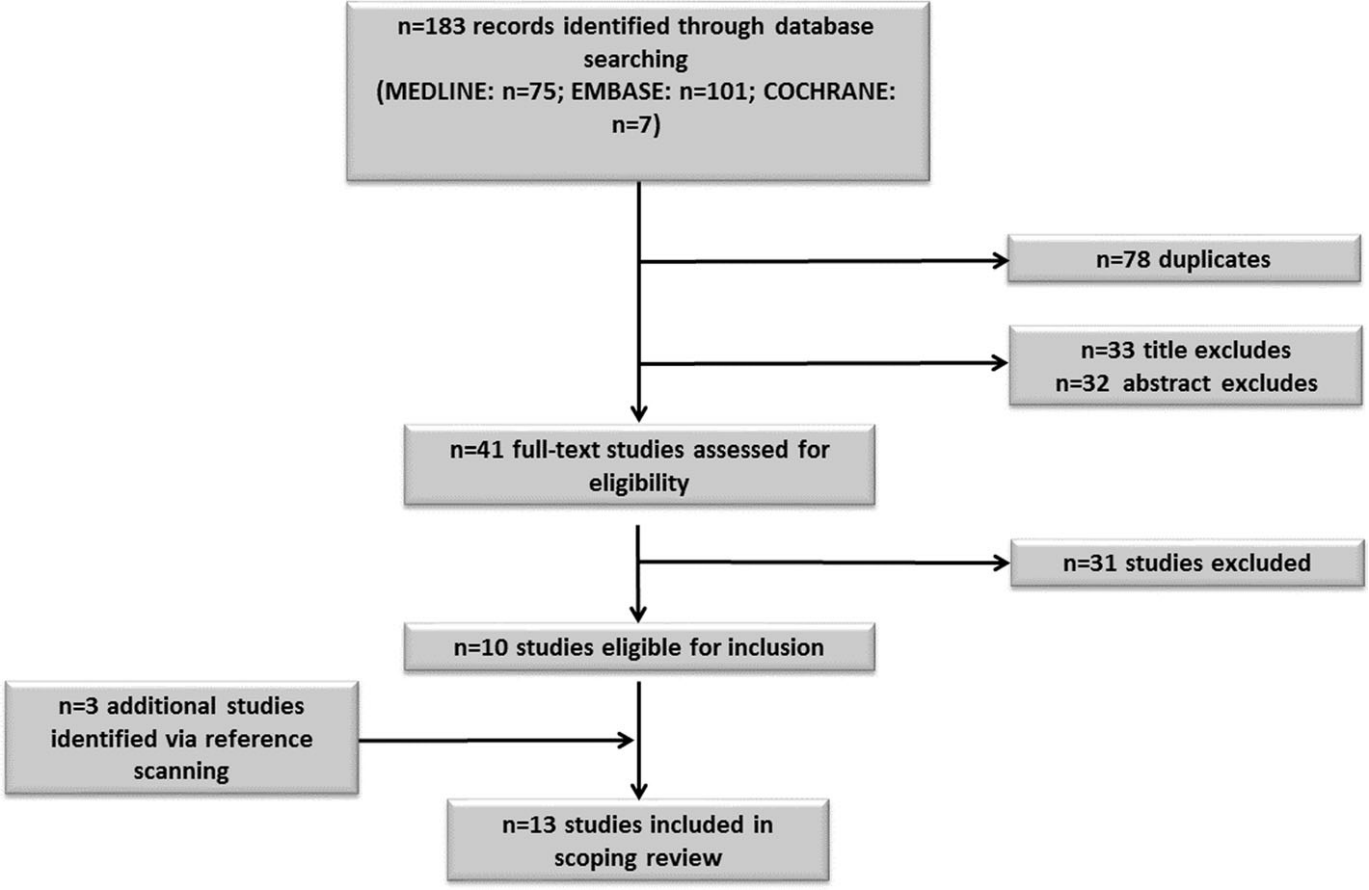
Study Flow Diagram

Following study selection thirteen papers were included in the scoping review: twelve observational studies and one randomised controlled trial (Fig. 1). The characteristics of the included studies are summarised in Table 1.

**Table 1:** Summary table of study characteristics

Author Year	Study Type	Follow-Up	Outcomes (not inc dislocation)	Dislocation	Recommendation
Paton and Hirst 1989 [27] UK	Observational	Min 6 months Max 4 years	–	PA: 8/93 (8.6%) DLA: 2/78 (2.6%) (*p* < 0.08)	DLA
Keene and Parker 1993 [28] UK	Observational	Up to 1 year	Operation duration Operative blood loss Perioperative fracture Subsequent fracture Superficial infection Deep infection Deep vein thrombosis Pulmonary embolism Sciatic nerve palsy Mortality Length of stay	PA: 10/229 (4.3%) DLA: 5/302 (1.7%) (***P*** **= 0.04)**	Surgeon choice
Unwin and Thomas 1994 [16] UK	Observational	3 months	–	PA: 149/1656 (9.0%) DLA: 41/1250 (3.3%) **(*p*** **> 0.001)**	DLA
Pajarinen et al. 2003 [29] Finland	Observational	6 months	–	PA: 14/86 (16.3%) DLA: 8/252 (3.2%) **OR 5.9 CI:2.4–15**	DLA
Enocson et al. 2008 [8] Sweden	Observational	0–10 years Median = 2.3 years	–	PA: 17/129 (13%) PA^a^: 15/176 (8.5%) LA: 13/431 (3%) ***p*** **< 0.001**	DLA
Ninh et al. 2009 [33] US	Observational	F-up 1: 6 weeks F-up 2: 1 year	–	PA: 9/139 (6.5%) DLA: 2/35 (5.7%) F-up 1: *p* = 0.069 F-up 2: *p* = 0.82	No recommendation
Biber et al. 2012 [32] Germany	Observational	Unclear	Early infection Early haematoma Early seroma Perioperative fracture	PA^a^: 3.9% DLA: 0.5% ***p*** **= 0.01**	No recommendation
Rogmark et al. 2014 [4] Sweden & Norway	Observational	Mean 2.7 years (SD(1.7))	Reoperation due to: Dislocation Infection Fracture Erosion and other	Total sample: ^b^443/33205 PA Hazard Ratio 2.2 (1.8–2.6)	DLA
Abram and Murray 2015 [10] UK	Observational	Up to 5 years 10 months	Infection Return home on discharge Mortality 30 day and 1 Year	PA^a^: 7/54 (13.0%) DLA: 16/753 (2.15) **p < 0.001**	DLA
Parker 2015 [34] UK	Randomised Controlled Trial	8 weeks 3 Months 6 Months 9 Months 12 Months	Pain Mobility Mortality Surgery length Patients transfused Units blood transfused Difficulty level Small/Large operative fracture Wound haematoma Wound infection (superficial and deep) Sciatic nerve palsy Later fracture around implant Re-operation General complications	PA^a^: 1/108 (0.9%) DLA: 2/108 (1.9%) *p* = 1.00	No recommendation
Leonardsson et al. 2016 [30] Sweden	Observational	1 year	Reoperation due to: -Infection -Fracture -Acetabular erosion Other Health related quality of life (patient reported) Pain (patient reported) Satisfaction (patient reported)	^b^PA: 20/978 (2%) ^b^DLA: 10/1140 (0.9%) ***p*** **= 0.02**	DLA
Kristensen et al. 2017 [18] Norway	Observational	4 months 12 months 36 months	Pain (patient reported) Satisfaction (patient reported) Quality of life (patient reported) Prosthesis survival	–	No recommendation
Svenøy et al. 2017 [31] Norway	Observational	1 year	Recurrent dislocation Infection (surgical site) Perioperative fracture Mortality (30 day and 1 year)	PA^a^: 15/186 (8%) DLA: 4/397 (1%)	DLA

^a^posterior repair explicitly noted by study authors

^b^figures only relate to dislocations requiring open reduction – no figures for dislocations treated by closed reduction provided

Figures in bold indicate a statistically significant difference in disocation rate between surgical approaches in individual studies

### Observational studies

Of the observational studies included four studies were UK based [10, 16, 27, 28], six were based in Scandinavian countries [4, 8, 18, 29–31], one was based in Germany [32] and one was from the USA [33].

There was a great deal of variation in the outcomes reported, ranging from a single outcome [16, 27] to over 15 [34]. Three studies used data from national registers [4, 18, 30]. Two observational studies included patient reported outcome measures; including pain, satisfaction and quality of life [18, 30]. Only one study did not report figures related to dislocation [18]. Dislocation rates ranged from 0.9 to 5.7% in DLA groups, and 0.9 to 16.3% in PA groups. Two studies examined the role of technical and patient related anatomical factors predisposing to dislocation using radiographs [29, 33]. Of the six studies reporting rates of surgical site infection (or reoperation due to infection), only one found a significant difference according to surgical approach; Keen and Parker [28] found a higher rate of infection using the DLA.

Overall eight observational studies recommended the DLA. All were based on findings related to dislocation. One study recommended that surgeons used their preferred approach or that with which they were more familiar [28], one study recommended subjective judgement based on potential severity of a range of complications [32].

### RCTs

The one randomised controlled study included in the review was conducted in the UK by a single experienced surgeon [34]. Regaining mobility was the primary outcome. A comprehensive set of secondary outcomes were also recorded. An objective measure of adductor function was performed with a subset of patients. This trial was halted before planned completion due to greater perceived technical difficulty using the PA. No significant differences in patient outcomes were found. No specific recommendation on surgical approach was made.

## Discussion

The aim of this review was to scope the primary research comparing the common surgical approaches used in hemiarthroplasty for treatment of displaced fractures of the femoral neck. The number of studies identified indicates that this is an area of practical concern to orthopaedic surgeons, however, it appears that the evidence base to inform guidelines remains limited. Published research is biased towards observational study designs. The recent emergence of cohort studies using data from large national registries is notable, but these are currently limited to Scandinavian countries. The heterogeneity of the included observational studies suggests that conducting a full systematic review would be of questionable value. Further randomised controlled trials, adequately powered to detect significant statistical and clinical differences in outcomes that are of interest to both patients and practitioners are needed.

One systematic review has previously addressed the issue of surgical approach for hemiarthroplasty [12]. This Cochrane review included only one RCT which compared the PA with an anterolateral approach technically different to the DLA [7]. The quality of this RCT was poor. Because of this the review authors were unable to draw a conclusion regarding an optimum surgical approach. In spite of this, this review has been used as one of the pieces of evidence informing the current NICE guideline (in addition to one other poor quality cohort study). A non-systematic narrative review by Rogmark and Leonardsson [35] also addressed this issue taking into account the results of Parker’s RCT and 2 other observational studies included in this review [8, 10]. The review recommended the direct lateral approach based on findings related to dislocation in these studies.

Although a thorough quality appraisal of the included studies is outside the scope of this review, it does appear that reporting standards are improved in more contemporary studies. Nonetheless, standardisation of reported outcomes is still not evident. Studies appear to differ on the variables collected and used to compare groups or perform multivariable analyses. Age and gender, and American Society of Anesthesiologists (ASA) physical status are almost universally recorded but other factors are included with mixed frequency, for example, experience of operating surgeons, cognitive status of patients, and duration of surgery. Other factors such as cementing and prosthetic type may also affect patient outcomes [4, 36]. Few studies using patient data from multiple sites restricted samples to a single implant type, or to cemented or uncemented femoral stems. Svenoy et al. [31] is a notable exception to this. Rogmark et al. [4] found an increased risk of reoperation for dislocation in patients with dementia. In studies where cognitive function was recorded, it tended to be by subjective assessment rather than the use of an objective measure. A set of core outcomes for hip fracture trials has been proposed [37] and its use in future studies would aid evidence synthesis.

Registry studies are valuable additions to the field, they utilise large samples and provide an accurate snapshot of outcomes on a national level. To recruit sufficient numbers to attain statistical power needed for investigating rare complications such as dislocation would be difficult in an RCT. However, as noted by Kristensen et al. [18], the increased risk of type-II error due to skewed distribution in surgical approaches must also be acknowledged. There remains a role for multiple research designs in addressing this issue. For outcomes such as pain and function RCTs are practical. As demonstrated by Parker, it is viable to compare surgical approaches using a randomised controlled methodology. An RCT offers the opportunity to control for factors such as cementing, prosthesis and surgeon experience [34]. On the other hand, the value of further small observation studies with clear risks of bias is questionable.

### Outcomes

The literature in this area has predominantly focused on dislocation as the primary outcome of interest. This is an unsurprising observation as the consequences of dislocation are devastating. Recurrent dislocation after the initial dislocation is common and multiple revision procedures are often required [8, 31]. Mortality post dislocation is extremely high. In the included studies there is a definite trend for higher rates of dislocation in patient groups operated on using a PA. A number of these studies, particularly those with low sample sizes should be treated with caution. It is unlikely that these studies were adequately powered to detect truly significant differences for a relatively rare outcome such as dislocation.

In spite of the focus on dislocation more contemporary studies consider pain and function as important outcomes. Clinicians appear to be paying more attention to patient reported outcomes, possibly reflecting calls to consider such outcomes in all modern surgical innovation. The single RCT included in this review used regain of function as the primary outcome; without significant difference between groups detected. With regard to patient reported outcome measures (PROMS) explored in observational studies Kristensen et al. [18] found less pain and better quality of life in the patients operated using the PA, but did not specifically recommend use of the PA over the DLA. Leonardsson et al. [30] found no differences in patient reported outcomes (HRQoL, pain and satisfaction) (adjusted analyses) and recommended surgical approach be based on risk of dislocation which was significantly higher in their study. The methods and samples used in both studies differ, but nonetheless the lack of consensus on PROMs indicates this area is not yet adequately explored.

### Terminology

The use of labels to describe surgical approaches to the hip capsule is often confusing [38]. Numerous approaches have been developed, and the same label has been used to describe distinctly different approaches. The majority of studies in this review used the term *direct lateral* to describe the Hardinge type approach. However a number used the term *anterolateral*, with or without an accompanying reference to Hardinge. Contacting authors was necessary to confirm the type of approach used. The term *anterolateral* is more suited to approaches that do not involve a direct dissection of the gluteus medius (for example, a Watson-Jones type approach). The field would benefit from consistent use of terminology. The majority of the reviewed studies provided only minimal description of surgical technique. Modifications to classic approaches are almost inevitable in modern usage. Modifications should be accurately described, using appropriate planar/morphological terms, and reference to the original technique.

### Limitations

This review has not addressed in detail additional factors that may affect outcomes in hemiarthroplasty of the hip. It is clear that a variety of prosthetic devices were used, both between and within studies, and there are some differences in post-operative care protocols, although these appear to be more consistent in more recent studies. The majority of the included studies emanated from either the UK or Scandinavia. Considering historical output in this area, this was expected. As studies not published in English were excluded, studies from other regions may have been missed.

## Conclusions

Current guidance on surgical approach for hemiarthroplasty is based on a limited selection of evidence. This scoping review has comprehensively assembled all of the relevant comparison studies in this area, and provides an overview of their characteristics. It has demonstrated that the evidence base remains limited in many respects. This poses a challenge to surgeons, systematic review of the currently available evidence is unlikely to provide a definitive consensus on which of the common surgical approaches provides for the best chance of functional recovery, balanced with acceptable risk of major complication. This issue would be best addressed by further RCTs, and high quality national-level observational data, with greater geographical representation than that currently available. The standardisation of recording patient risk factors, detailed surgical and post-operative intervention protocols and outcomes in all study designs would strengthen the potential for valid comparison of findings.

## Abbreviations

DLA: Direct lateral approach
PA: Posterior approach
